# Rifampicin resistance in new bacteriologically confirmed pulmonary tuberculosis patients in Cameroon: a cross-sectional survey

**DOI:** 10.1186/s13104-018-3675-0

**Published:** 2018-08-13

**Authors:** J. Noeske, A. Nana Yakam, J. L. Abena Foe, D. Nguafack, C. Kuaban

**Affiliations:** 1 Douala, Cameroon; 2 University of Douala, PoBox 4032, Douala, Cameroon; 3 Yaounde, Cameroon; 4 Focal Point MDR-TB, National Tuberculosis Program, Yaounde, Cameroon; 5 Department of Medicine, University of Bamenda, Bamenda, Cameroon

**Keywords:** Bacteriologically confirmed tuberculosis, Rifampicin resistance, New cases, Cameroon

## Abstract

**Objective:**

In Cameroon, tuberculosis (TB) cases are diagnosed and treated within a nationwide network of 248 diagnostic and treatment centres. In 2016, the centers notified a total of 175 multidrug-resistant (MDR-)TB cases, most of them retreatment cases. According to the WHO, the expected number of MDR-TB cases was estimated to be 1200 (1000–2200) corresponding to a rate of 6.8 (4.3–9.4) per 100,000 population. This indicates a notification gap of more than 80%. The objective of this study was to estimate the prevalence of MDR-TB in new bacteriologically confirmed pulmonary TB cases. We undertook a nationwide cross sectional survey during 6 weeks.

**Results:**

During the study period, the NTP notified 1478 new bacteriologically confirmed pulmonary TB cases. Among them, 1029 (70%) had a valid Xpert result and 16 were identified with rifampicin resistant (RR-TB), a tracer of MDR-TB. This gives a prevalence of 1.6% (95% CI 0.8–2.3) among incident cases. The rate of RR-TB in the regions varied between 0 and 3.3%. If the results of this study are confirmed, the incidence rate given by WHO (2.8%, 95% CI 2.1–3.4) might be an over-estimation.

## Introduction

**Fig. 1:**
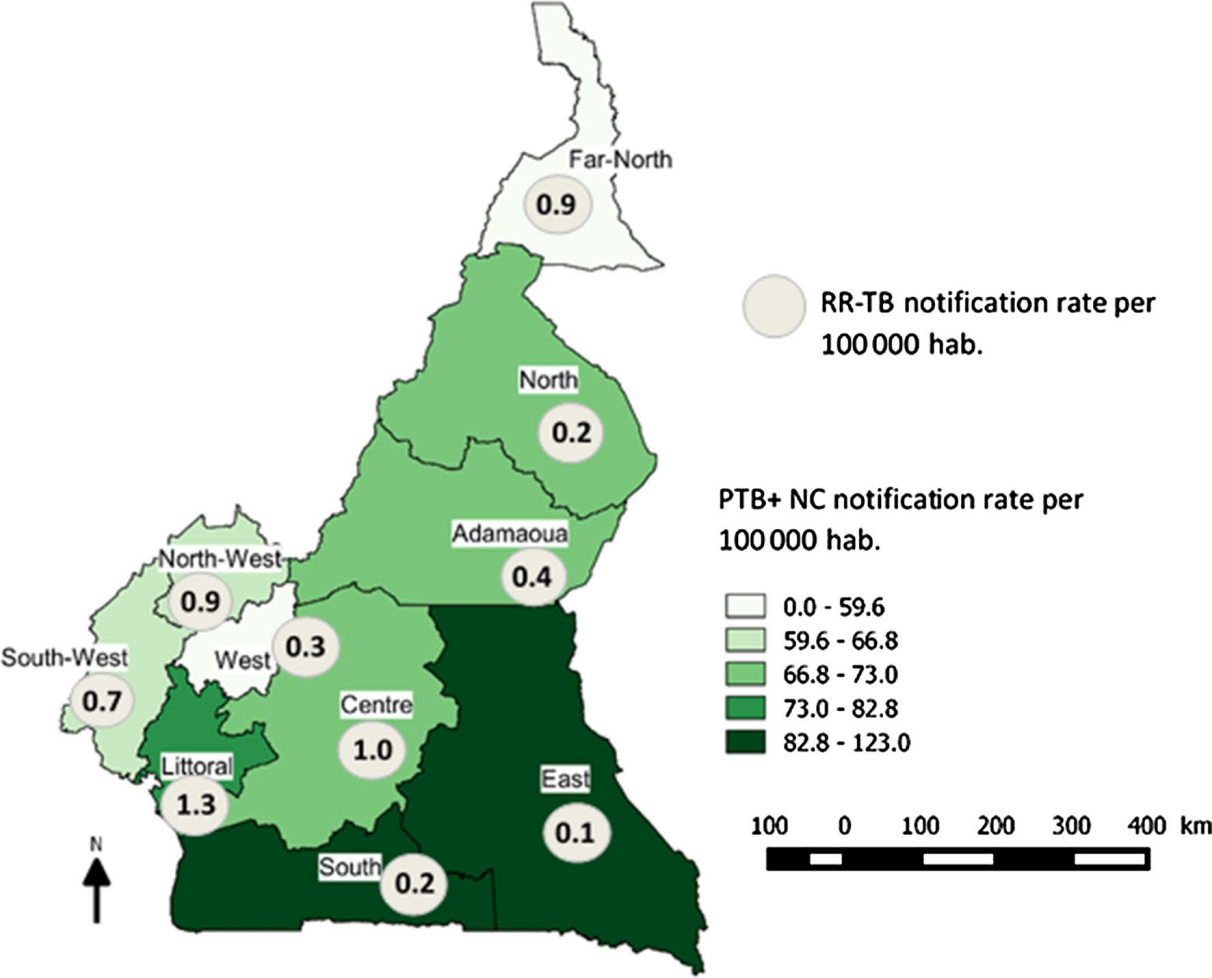
Notification rates of new bacteriologically confirmed pulmonary tuberculosis (all and rifampicin resistant cases) per 100,000 population and per region, Cameroon 2017

In Cameroon, virtually all tuberculosis (TB cases) are diagnosed and treated within a nationwide network of 248 tuberculosis diagnostic and treatment centres (DTC) managed by the National TB Programme (NTP). In 2016, the DTCs notified a total of 25,975 TB cases of which 175 were rifampicin resistant and/or multidrug resistant (RR/MDR-TB) patients [1, 2]. The map displays TB notification numbers and rates per region (Fig. 1). According to the WHO estimate, the number of TB incident cases, susceptible as well as RR/MDR-TB, for Cameroon are far higher than the number notified. In 2016 the WHO estimated the number of RR/MDR-TB incident cases to be 1200 (1000–2200) corresponding to a prevalence rate of 6.8 (4.3–9.4) per 100,000 population. This indicates that there is a notification gap of more than 80% [1]. The large confidence interval around the WHO estimate however indicates the level of uncertainty in the figures derived from mathematical modeling. Following the recommendations of WHO, previously treated cases as well as TB contacts of RR/MDR-TB are systematically tested in Cameroun. Coverage of previously tested cases—about 1500 per year—attains almost 80%, allowing the NTP to estimate at 14–16% the prevalence of RR/MDR-TB in this patient group, thus expecting a total of 225 cases per year. The question that can be asked is whether the gap observed between the number of RR/MDR-TB incident cases estimated by the WHO and the number effectively notified by the NTP can be covered by testing new bacteriologically confirmed pulmonary TB cases (PTB+ NC). Indeed, nothing is known about the magnitude of RR/MDR-TB incident cases among this patient group. Microscopy, the main diagnostic tool for TB in new cases in Cameroon does not allow for diagnosing drug resistant TB. It is for this reason that the NTP undertook a nationwide cross sectional survey to get a more accurate estimate of RR/MDR-TB prevalence for Cameroon among new pulmonary TB cases.

## Main text

### Materials and methods

The study was performed in all the 248 DTCs in the ten regions of Cameroon over a period of 6 weeks (13th of March to 21st of April 2017). All patients aged 15 years and above presenting at each DTC with symptoms compatible with pulmonary TB were asked according to NTCP guidelines to submit two sputum samples which were examined microscopically [3]. A patient with a positive sputum for acid-fast bacilli (AFB) who had never been treated with anti-tuberculosis drugs for more than 1 month was considered a new pulmonary TB case with bacteriological confirmation (PTB+ NC). Patients previously treated for TB as well as those with uncertain TB antecedents were excluded from the study.

TB laboratories of the DTCs were asked to keep after the microscopic examination the remaining positive sputum specimen for each patient in a fridge or a cold place and to send it within 3 days through the Regional TB Control Unit to one of the two National TB Reference Laboratories (TRL) in the country. In the TRL, the sputum sample for each patient was tested for rifampicin resistance using Xpert^®^MTB/RIF assay (Cepheid, Sunnyvale CA, USA). Positive sputum specimens on the first examination were confirmed by a second Xpert test. For positive sputum specimens that gave an indeterminate test result, the Xpert test was repeated again. Patients identified as PTB+ NC in each DTC were put without delay on the 6-month standard anti-tuberculosis regimen according to the NTP guidelines. This regimen was changed to the standard 9-month short course MDR-TB regimen for patients identified with RR-TB as per NTCP guidelines as soon as the valid RR results were communicated to the respective DTCs [2].

Estimating the prevalence of RR/MDR-TB at 2.5% among new PTB cases and aiming at a confidence interval of 95% with a precision of ± 1% and using the sample size formula for the estimation of a population proportion [4], the sample size for the study was calculated to be 1000 sputum smear positive PTB patients. Taking into account the yearly rhythm of TB notifications from the DTCs, the study period was fixed to last 6 weeks.

### Results

During the study period, a total of 1478 PTB+ NC were diagnosed by the DTCs. Coverage of the study accessible population varied from 45 to 100%, five out of ten regions comprising 45% of the population having a coverage of ≥ 85%. A total of 1029 PTB+ NC (70%) had a valid Xpert result. Sixteen patients were identified with RR-TB giving a prevalence of 1.6% (95% CI 0.8–2.3). The rate of RR-TB in the regions varied between 0 and 3.3% (Table 1).

**Table 1:** Number (%) of new bacteriologically confirmed pulmonary tuberculosis cases tested and identified with rifampicin resistant TB, Cameroon 2017

Region	PTB+ NC notified	Number (%) PTB+ NC sampled	Number (%) PTB+ NC with RR notified	95% CI proportion PTB+ NC with RR
Littoral	241	212 (88)	2 (0.9)	0–2.2
NW	159	103 (65)	2 (1.9)	0–4.6
West	71	71 (100)	1 (1.4)	0–4.1
Est	97	61 (63)	0	–
Ada	95	83 (87)	1 (1.2)	0–3.6
Nord	141	120 (85)	4 (3.3)	0.1–6.5
EN	211	95 (45)	2 (2.1)*	0–5.0
CE	283	136 (48)	2 (1.5)	0–3.5
Sud	88	66 (75)	0	–
SW	92	82 (89)	2 (2.4)	0–5.8
Pays	1478	1029 (70)	16 (1.6)	0.8–2.3

* Test result of patients not confirmed by a second Xpert exam

### Discussion

This paper presents the first evidence-based estimation of the magnitude of RR-TB among new bacteriologically confirmed cases of pulmonary tuberculosis in Cameroon. Hitherto, estimates principally proposed by WHO were derived from mathematical modeling [1]. If the results of our study are confirmed, the incidence of RR/MDR-TB in new PTB+ cases in Cameroon may have been overestimated.

At least, three preliminary conclusions can be drawn from these results. Firstly, it might be stated that the RR/MDR-TB situation has not attained “epidemic or generalized” dimensions in Cameroon. Strains might still be circulating rather in circumscribed areas or in groups of individuals, a phenomenon corroborated by the anecdotal observations of 25–35% of newly diagnosed RR/MDR-TB found in “clusters”. Another conclusion concerns strategic planning by the NTP. The program’s resources are limited. Universal access to rapid testing for MTB and rifampicin resistance of all presumptive TB cases as foreseen by the end-TB strategy by the end of 2020 is out of reach for the program because of financial barriers. While Xpert testing of candidates for TB retreatment who belong to a high risk group of RR/MDR-TB is sound for programmatic management, the question is how to handle programmatically under the given circumstances of financial constraints presumptive RR/MDR-TB in new cases. Xpert testing is already however carried out for microscopically unconfirmed pulmonary TB cases with some RR/MDR-TB detected albeit in small proportions. From a strategic point of view, two approaches for the diagnosis of RR/MDR-TB in new cases seem promising. One of these is a systematic comprehensive screening or investigation of RR/MDR-TB contacts which as far as possible should be repeated at least once after a 6 month period [5–7]. The other approach may be GPS mapping of all RR/MDR-TB cases and extending Xpert testing to groups of persons belonging to “hotspot” areas or identified risk groups from which the source case came; for example testing all presumptive TB cases in prisons from where RR/MDR-TB cases have been notified. Finally, the survey demonstrated that even with a limited network of diagnostic facilities for RR/MDR-TB, nationwide coverage of all presumptive cases by molecular testing is feasible.

### Conclusion

A cross-sectional survey among new bacteriologically confirmed pulmonary TB cases in Cameroon revealed a prevalence of 1.6% (95% CI 0.8–2.3) RR-TB among this patient group, an percentage significantly lower than the one given by WHO estimates. Although the results of the survey have to be interpreted with caution because of possible biases, they help inform the NTP for conceiving strategies to control RR/MDR-TB in Cameroon.

## Limitations

Yet, the results of the study have to interpreted with caution. Firstly, coverage of the target population to be sampled and tested did not exceed 70%. Although we achieved the sample size for the study, DTCs in some regions notably the Centre and Far North Regions did poorly in enrolling study subjects due to organizational difficulties. This resulted consequently in all DTCs not succeeding in sampling all consecutively diagnosed PTB+ NC over the study period. This might have therefore introduced some selection bias. However, during evaluation sessions with DTCs in seven of the ten regions and comparing name by name notified with samples patients no systematic exclusion (series, groups of) patients were noted.

## Abbreviations

AFB: acid fast bacilli
CI: confidence interval
DTC: Diagnostic and Treatment Center
GPS: global positioning system
MDR-TB: multidrug resistant tuberculosis
MTB: *Mycobacterium tuberculosis*
NC: new case
NTP: National Tuberculosis Program
PTB+: bacteriologically confirmed tuberculosis
RR: rifampicin resistance
TB: tuberculosis
TRL: Tuberculosis Reference Laboratory
Xpert: GeneXpert MTB/RIF
WHO: World Health Organization
